# The Total Dietary Antioxidant Capacity, Its Seasonal Variability, and Dietary Sources in Cardiovascular Patients

**DOI:** 10.3390/antiox12020292

**Published:** 2023-01-28

**Authors:** Magdalena Czlapka-Matyasik, Anna Gramza-Michalowska

**Affiliations:** 1 Department of Human Nutrition and Dietetics, Poznan University of Life Sciences, 60-624 Poznań, Poland; 2 Department of Gastronomy Science and Functional Foods, Faculty of Food Science and Nutrition, Poznan University of Life Sciences, 31 Wojska Polskiego Str., 60-624 Poznań, Poland

**Keywords:** total diet antioxidant capacity, ORAC, seasonal variation, cardiovascular patients

## Abstract

The favourable role of dietary antioxidants in cardiovascular diseases (CVDs) and protection from them is widely discussed, and total dietary antioxidant capacity (TAOX) is perceived as a diet-quality marker. Data concerning TAOX and its dietary sources related to seasonal variability are limited. We aimed to analyse the TAOXs, seasonal variability, and sources in the daily diets of CVD patients. A total of 143 subjects (82 men, 61 women) since CVD problems were studied. Seasonal recalls were collected regarding dietary sources of antioxidant compounds in spring, summer, autumn, and winter. A food frequency questionnaire was used. The total dietary antioxidant capacity (in μmolTE/day) was calculated for each season. The primary sources of antioxidants in cardiovascular patients’ diets were drinks (33%), fruits (28%), vegetables (16%), and black tea (14%). The TAOXs of CVD patients’ diets significantly depended on the season (*p* < 0.001) and were highest in the summer and lowest in the spring. This seasonal variation in consumption was noted. Our findings suggest that a diet characterised with a TAOX might be subjected to fluctuations between seasons. We suggest considering modifications in the dietary recommendations for cardiovascular patients with a low antioxidant capacity between seasons.

## 1. Introduction

Total dietary antioxidant capacity is generating considerable interest for its healthy properties. Its beneficial role is discussed particularly in terms of civilisation diseases. It has been shown that increased antioxidant capacity might be related to reduced risk of hypertension, selected cancers, glucose tolerance, and central adiposity [1,2,3,4,5,6]. Bearing in mind that according to a WHO report, CVDs are responsible for 31% of all global deaths each year, constituting the leading causes of death worldwide, studies of factors that prevent CVDs are drawing significant attention around the world [7]. The search for new markers of diet quality associated with lower CVD risk has become the subject of many studies. Thus, the favourable role of dietary antioxidants in CVD has been widely discussed, and in the last decade, dietary antioxidant capacity has become the most debated parameter [8,9,10,11,12]. Evidence that increased diet antioxidant capacity might present promising benefits in CVDs has been postulated in the literature [10,11,13,14].

Regardless of several reviews in the literature that address the importance of diet antioxidant capacity, none of the recently published articles has comprehensively discussed doses of diet antioxidant capacity for CVD patients, its dietary sources, and variability between seasons [2,15,16].

In science, antioxidant capacity has been considered from many perspectives: for example, as a measure of oxidative stress for analysis of oxidative stress markers in body fluids. This process’s sensitivity and validity have been discussed due to various inflammatory reactions involved in oxidative stress in numerous biological conditions. Moreover, antioxidant capacity accompanies the foods consumed in a daily diet. Its levels testify to the potential abilities of food to counteract oxidative stress. So far, in many studies, high antioxidant properties have been demonstrated in selected foods [3,17,18,19,20], and antioxidant capacity measured through different methods and techniques has been discussed widely [21,22,23]. Another critical measure is the total antioxidant capacity of the diet (TAOX), measured in ORAC, FRAP, ABTS, or other units. Knowledge concerning the TAOXs of the whole foods consumed in a daily diet would give a chance to estimate preventive and therapeutic recommendations in nutrition. Knowledge of the antioxidant-capacity distribution between food groups, which may differ in many climates and seasons, would be beneficial in this. This climatic variability leads to changes in the maturity and availability of foods, and thus, changes in antioxidant capacity should also be monitored. According to Pacifico et al., for example, changes in the bioactivity of wild rue related to the harvest season gained 44% TAOX [24].

Many authors have pointed to the necessity to consider the diet as a whole conveyer of antioxidant capacity. Association of less-atherogenic blood profiles with greater antioxidant capacity from diet and supplements was shown in U.S. adults [25,26]. The top contributors to TAOX were tea, antioxidant supplements, vegetable mixtures, orange juice, berries, and wine. Another study, by Okudo et al., showed that fruits, vegetables, tea, and coffee were the main contributors to dietary total antioxidant capacity [5]. Its positive relation to frailty among elderly Japanese women was indicated by Kobayashi et al. [27].

Beneficial antioxidant properties against cardiovascular diseases have been indicated in reviews [13,14,16,28,29]. Their summaries have confirmed that the increased supply of antioxidants is associated with reduced CVD.

Thus, antioxidant capacity has become a notable diet quality marker. It is widely known that measurement is highly appreciated in diets, but methods, procedures, and standardisation are needed. This issue is still open and widely discussed.

Nevertheless, data concerning total dietary antioxidant capacity (TAOX) and its dietary sources related to seasonal variability, especially in CVD patients, are limited and differ. Despite many published results that have discussed the benefits of dietary antioxidants, their doses and unified units are unknown. The recommended daily intake has not yet been determined, its seasonal variations have not been discussed, and dietary sources in the central European diet have not been comprehensively presented. To address these gaps in the study outlined above, we formulated research questions related to seasonal variation of dietary antioxidants and total dietary antioxidant capacity supply in CVD patients.

## 2. Materials and Methods

### 2.1. Patients and Methods

The recruitment process was conducted at the cardiology department of the State Clinical Hospital in Poznan. The data for this cross-sectional study were collected in the Department of Human Nutrition and Dietetics, Poznan University of Life Sciences. The inclusion criteria were age of 20 to 90 years and cardiovascular events that included a myocardial infarction over the last five years prior to hospitalisation. The exclusion criteria were failure to meet the inclusion criteria; any dieting, including weight-reduction therapies; food allergy or food intolerance, kidney or mental disease, and taking of dietary supplements. Initially, 150 cardiovascular patients were invited to attend. Finally, 143 cardiovascular patients (82 men, 61 women), aged 61y on average (23–89), completed interviews (Table 1). In the first stage, anthropometric parameters and body composition were determined, and then information on food intake was obtained through direct interviews. Each subject was examined according to previously specified procedures [30]. All information was collected through “face–to–face” contact. Well-trained, qualified dieticians conducted the investigations and measurements.

A validated semiquantitative food frequency questionnaire with a photo album of dishes was used [31,32]. Subjects were asked to indicate intake frequency of and sizes of daily portions. Before starting the examination, respondents were not given any information concerning proper nutrition.

The total diet antioxidant capacity in ORAC (oxygen radical antioxidant capacity in μmolTE/day) was calculated for each season (spring, summer, autumn, and winter) and in total during the entire year by summing up the ORAC values of foods according to the USDA database 2011 [33]. Analytical evaluations of ORAC were performed for foods that made up components of a typical Polish diet but were not included in this USDA database, using methods consistent with the American database.

The following values were calculated:

(A) Total diet antioxidant capacity (T-ORAC) (in μmolTE/day);
(B) Total antioxidative density of diet (Q–ORAC), i.e., antioxidative potential per 1000 kcal of diet (in μmolTE/1000 kcal) [34];
(C) Shares (%) of selected food groups in total antioxidative density, distinguishing 19 food groups: (1) total drinks; (2) fruits (gooseberry, pineapple, watermelon, avocado, banana, peach, lemon, grapefruit, pear, apple, blackberry, kiwi, raspberry, tangerine, mango, melon, apricot, nectarine, orange, currant, plum, strawberry, grape, cherry); (3) vegetables, incl. potatoes (eggplant, broccoli, beetroot, onion, garlic, pumpkin, cauliflower, white cabbage, red cabbage, maise, carrot, cucumber, pepper, parsley, potato, tomato, leek, radish, lettuce, celery, chive, spinach); (4) black tea; (5) cocoa; (6) green tea; (7) potatoes; (8) dried fruits (date, fig, apricot, dried plum, raisin, cranberry); (9) fruit juices; (10) herbs and spices (basil, curry, cinnamon, nutmeg, clove, ginger, marjoram, oregano, pepper, parsley, sage, thyme); (11) grains; (12) leguminous plants (white dry bean, string bean, green pea, lentil, soya bean); (13) nuts (almond, pistachio nut, walnut, hazelnut); (14) total wines (all types); (15) vegetable juices; (16) red wines; (17) olive oil; (18) chocolate in total (all types, e.g., candy bar, leavened cake, various types of chocolate); and (19) bitter chocolate.
(D) The total daily diet nutritional value, adjusted per 1000 kcal. To accomplish adjusted values called “nutrient densities”, micronutrient values were expressed as intake (in appropriate units)/1000 kcal. Energy adjustment is advantageous in analyses of diet–disease associations and is used when a food frequency questionnaire (FFQ) is the main dietary assessment instrument [35].

### 2.2. Statistical Analysis

The Clinical Calculator (ClinCalc, LLC) was used to calculate the sample size [36]. Sample size calculation was based on means and standard deviations of total antioxidant density via Q-ORAC in μmolTE/1000 kcal. A database from a previously published survey that used an FFQ that covered 48 adults was used [30]. The assumption of a two-sided significance level of 0.05 and 85% power was used to detect a 20% difference in the mean values of the scores between the winter and summer data. The minimum sample size was 130, including a 10% dropout rate and 10% missing data. The calculation results were compared with those of similar validation studies [37] and a literature review by Cade et al. [38]. 

Continuous data were presented as means with 95% confidence intervals (95% CIs), medians (minimum, maximum), and categorical variables as sample percentages (%). Differences between groups were verified using a two-tailed t-test (continuous data) or an χ^2^ test (categorical data). Variable normality was checked using a Kolmogorov–Smirnov test. 

The following tests were used for the verification of differences between groups:

- T-test for independent samples, men and women (Table 1.),
- Friedman ANOVA based on rank for repeated measurements to compare Q–ORAC of subjects’ diets in 4 seasons (Table 2); and shares (%) of selected food groups in the total Q–ORAC of these subjects’ diets in 4 seasons (Table 3).

Statistical analysis was carried out using StatSoft, Inc. STATISTICA 13.3; *p*-values < 0.05 were considered statistically significant.

## 3. Results

### 3.1. Sample Characteristics

The mean age of the study sample was 61 years old (Table 1). Most participants lived in urbanised areas (76%) and declared a secondary education level (72%). Considering employment sex, men were more often employed than were women (40 vs 25%), while 72% of women vs men (56%) were pensioners or retired.

### 3.2. Total Dietary Antioxidant Capacity (TAOX) and Nutritional Value of Daily Diet

The total dietary antioxidant capacity and daily dietary intake mean values are presented in Supplementary Material (Table S1). No significant difference in T-ORAC intake between men and women was revealed (25,174 and 26,238 μmolTE/day, respectively). The daily antioxidant density (Q-ORAC/1000 kcal) was significantly lower in men than in women (12,797 vs. 16,521 µmolTE/1000 kcal). Adjustment of dietary intake revealed that men’s diets were significantly lower sources of total proteins (*p* < 0.05), plant proteins (*p* < 0.01), total carbohydrates (*p* < 0.05), potassium (*p* < 0.01), calcium (*p* < 0.001), phosphorus (*p* < 0.01), magnesium (*p* < 0.01), iron (*p* < 0.01), manganese (*p* < 0.05), vitamin A (*p* < 0.001), beta-carotene (*p* < 0.001), vitamin E (*p* < 0.01), thiamine (*p* < 0.05), riboflavin (*p* < 0.01), vitamin B6 (*p* < 0.001), folate (*p* < 0.001), vitamin C (*p* < 0.001), and fibre (*p* < 0.001). Women’s diets were significantly lower sources of total fat (*p* < 0.05), sodium (*p* < 0.05), and MUFAs (*p* < 0.05).

### 3.3. Shares of Selected Foods and Seasonal Variability in Total Dietary Antioxidant Capacity

The primary TAOX sources in the total group diets were drinks (32.9%); fruits (27.6%); vegetables, including potatoes (16.5%); black tea (14.4%); cocoa (13.3%); and green tea (5.3%) (Table 2). Foods that contributed less than 5% of the total capacity of the diet were dry fruits, fruit and vegetable juices, herbs and spices, grains, legumes, nuts, wines, oils, and chocolate. We did not observe differences between men and women except for in herbs and spices and in vegetables, where women’s diets were the higher source of antioxidant capacity. 

The shares of food groups such as total drinks, fruits, vegetables, and black tea in the Q–ORAC of the total group diets significantly depended on the season. The primary source of TAOX in the spring, autumn, and winter was drinks, while in the summer, it was fruits (Table 3).

## 4. Discussion

Our study revealed the total dietary supply of natural antioxidants in cardiovascular patients. We revealed that the men presented decreased total dietary antioxidant density compared to the women. Interestingly, significant variations in the seasonality and shares of the dietary sources of natural antioxidants were observed.

Studies regarding total dietary antioxidant capacity (TAOX) in cardiovascular patients are limited, to the best of the authors’ knowledge. Therefore, it is not easy to compare our results to the data in the literature. An additional difficulty is the variety of units of expression of levels of natural antioxidants used to evaluate the antioxidant capacities of foods. There has not been a consensus as to the preferred method. ORAC (oxygen radical absorbance capacity), Trolox equivalent antioxidant capacity (TEAC), total radical-trapping antioxidant parameter (TRAP), and ferric reducing antioxidant power (FRAP) are among the more popular methods that have been used. Despite similarities and correlations, not all of the measures mentioned can be compared and discussed directly as markers of oxidative stress in organisms. Instead, many authors have suggested that dietary antioxidant supply should be considered the measure of diet quality, modifying the risk factors responsible for cardiovascular, cancers, mortality, and diabetes, which has already been confirmed in many studies [1,2,5,8,28,29]. Bearing in mind the availability of sources and the possibility of performing ORAC analyses of local products of the typical Pole’s diet, we decided to use USDA ORAC data as a starting point to calculate the antioxidant potentials of diets in this study [33]. We want to emphasise that this base includes typically American products. Therefore, using the same methodology, we made a series of analyses (unpublished data) of typically Polish products to calculate the total dietary antioxidant intake. 

We noted that TAOX depended on the study group’s dietary habits. Its variability is explained through cooking methods, seasonality of consumption, and diet structure related to the amount of plant and animal sources in daily intake. A wide range of TAOX values is published in the literature. Hervert-Hernández et al. (2011) presented the Mexican rural diet of 1000–2000 µmol TE/day and justified this low daily intake of fruits and vegetables [39]. The total mean antioxidant capacity of the Spanish diet presented by Gonzales et al. was between 5873 and 11,263 µmolTE/day [40]. 

Finally, the highest TAOX was presented in the Korean diet and showed 54,335 μmolTE/day [41]. It must be pointed out that the intake of cereals, fruits, and vegetables in the Asian diet is the major contributor to its TAOX, next to dietary habits preferred in the population. Our previous study showed that the TAOX of young women was 18,661 TE/day/1000 kcal and differed significantly between seasons [30]. The estimation of TAOX in this study was based on the use of foods consumed in the diet. Data were collected by qualified dieticians via a semiquantitative validated FFQ and included seasonal variability. We revealed that cardiovascular patients consumed 25,628 μmolTE/day. Such a result seems reasonable, especially if we compare this level to the 29,006 μmolTE/day recommended by Martinez et al. in the 2000 kcal diet [42]. Our results are in line with those obtained by Iranian authors regarding pregnant women (20,284 μmolTE/day) and 60-year-old Swedish women (16,288 μmolTE/day) [37,43]. We assume that the TAOX supply in the daily diet should be diversified and targeted to age, health risks, and prevention. Despite many reports supporting this hypothesis, the exact doses of antioxidant capacity among cardiovascular patients are rarely determined.

Another aspect of the daily diet’s beneficial properties is its ‘nutritional density’, defined as the number of beneficial nutrients in a food product in proportion to, e.g., energy content. The application of nutritional density in supporting consumers in identification of foods that provide optimal nutrition was underlined previously and has been widely used in the literature [44,45,46,47]. Therefore, to standardise levels of TAOX in the present study, data were adjusted for 1000 kcal of energy (Table S1). Energy adjustment is the method in which nutrients are evaluated in relation to total energy intake. Our study additionally used it to mitigate the effects of measurement error in data collected using self-reported dietary assessment instruments.

After adjustment, men’s diets were a 31% lower source of natural antioxidants than were women’s, and those differences were statistically confirmed. Following those, we analysed nutrient density. We concluded that men’s diets had lower density regarding potassium, calcium, phosphorus, magnesium, iron, manganese, vitamin A, beta-carotene, vitamin E, thiamine, riboflavin, vitamins D and B6, folate, vitamin C, and fibre. In contrast, the same diet had a significantly higher total fat and sodium density. Such results gave us the background to indicate that men’s diets characterised significantly higher atherogenic effect than women’s.

In the present study, we determined the contributions of different food groups to total antioxidant intake. The T-ORAC was mainly derived from drinks (~33%), fruits (~28%), and vegetables (~16%). While we expected a significant plant-food contribution to the TAOX, we were surprised to observe that drinks were the major contributor to the total intake of antioxidants. High antioxidant levels in drinks were reported in several studies [48,49,50,51], but this high contribution to total dietary intake of antioxidants was noted and discussed in only one study before [52]. Some authors indicated a high coffee contribution to the TAOX in a group of native Norwegians [52].

Contrary to what was reported by Savillas et al. in the Norway diet, our results indicated the main contributions in TAOX were black tea and cocoa (~14%). To explain those data, we indicate the traditional dietary patterns of 60y Poles, in which tea is the most preferred beverage [53]. Moreover, our subjects had already cardiovascular events and were treated in the cardiac clinic, where the recommendation of coffee limit since hypertension risk was applied. 

In summation of the dietary habits of the study sample in our research, it must be pointed out that dietary intake and consumption of food groups are related to many factors. Those relations and influences, including family influence [54] and knowledge and perception of nutritional benefits [55], were indicated in our previous papers. 

Two groups significantly differentiated men and women in contribution to total antioxidant intake. We discovered that vegetables (16 and 18%) and herbs and spices (1.4 and 2.6%) were sources of natural dietary antioxidants. Several studies have addressed factors that underlie gender-specific food-choice patterns [56,57,58]. In a study of older UK adults, aged 55–64 y, men estimated the recommended portions of fruit and vegetables to be lower than did women [59]. Our outcomes related to identifying differences in consumption of herbs and spices were in line with previous studies that reported a lower preference for herbs and spices by men. [46,59,60,61]. It has been shown that food choices, including of spices, are explainable with gender preferences [62,63]. In our study, men’s preference for consumption of spices, i.e., basil, curry, cinnamon, nutmeg, clove, ginger, marjoram, oregano, pepper, parsley, sage, and thyme, was lower than that of women.

The most striking observation to emerge from this analysis was the seasonality of consumption. Consequently, diet antioxidative density significantly depended on the season. In our study, the highest T-ORAC was noted in the summer, before the autumn, the winter, and the spring. In the summer, we observed a ~55% higher TAOX than in the spring. 

It is important to point out that those variations between seasons were noted in drink; fruit; vegetable, including potato; and black tea intake. Such results are consistent across nations and ages [64,65,66,67,68]. The outcomes we obtained were in line with our expectations. The summer season in Poland is the period of the greatest availability of fresh vegetables and fruits, most often consumed in this form. Winter and spring are transitional periods when imported fruits and vegetables are available on the market and consumed less readily. Although fresh vegetables are available in spring, they are consumed much less frequently and eagerly. Their consumption is limited by purchase price and limited confidence in their nutritional value.

The seasonal diversification of consumption in the literature shows the prevalence of this phenomenon, regardless of country and other factors that regulate consumption [66]. In addition, it shows the need to diversify seasonal recommendations for dietary intake.

Although this research reached its aims, we are aware that it may have some limitations. First, although the minimum sample-size criteria were met in this study, our investigations were only on a small scale. A larger sample size would reveal more significant differences between groups, allowing stronger conclusions. 

Second, it could be that CVD patients are more prone to omit food inputs due to concerns about how they would be perceived if they reported input of foods that were incongruent with dietary recommendations. However, the aim of this study was to analyse CVD subjects’ intakes; our results are limited to this population and may not apply to the younger and healthier population.

Altogether, what stands out is that seasonality differences should be considered in dietary recommendations for populations at risk of cardiovascular disease. 

## 5. Conclusions

To the best of our knowledge, this research is the first study to identify total dietary antioxidant capacity in CVD patients. Our findings suggest that a diet characterised with a TAOX might be subjected to fluctuations between seasons and provide key information to choose the most appropriate dietary sources of antioxidants. 

Although summer as a season proved to be the best time in the supplies of natural antioxidants and their best source, juices and fruits, gender differences in food preferences in favour of women were stark. 

We would like to underline that dietary recommendations, including an increased supply of natural antioxidants, should correct the significantly higher atherogenic character of men’s diets. We suggest consideration of modifications in the dietary recommendations for cardiovascular patients in low-antioxidant-capacity seasons, including consumption of natural juices as carriers of antioxidant capacity. 

More studies are needed to confirm the causality and explore the role of foods rich in antioxidants in prevention of CVD. Prospective and retrospective trials are an urgent calling to confirm this conclusion.

## Figures and Tables

**Table 1:** Sample characteristics ^1^.

Variable	Men + Women (n = 143)				Men (n = 82)				Women (n = 61)				*p* ^3^
	X ^4^ (95%CI)	Me ^5^	Min. ^6^	Max. ^7^	X (95%CI)	Me	Min.	Max.	X (95%CI)	Me	Min.	Max.	
Age (Years)	61 (59; 63)	61	23	89	60 (57; 62)	61	23	83	63 (61; 66)	61	46	89	NS
Weight (kg)	83.6 (81.1; 86.2)	82.8	50.0	136.0	89.5 (86.4; 92.5)	90.0	60.0	136.0	75.9 (72.5; 79.4)	76.5	50.0	109.0	***
BMI (kg/m^2^)	29.3 (28.5; 30.1)	29.1	19.0	44.8	29.2 (28.3; 30.1)	29.1	19.4	43.0	29.4 (27.9; 30.8)	29.7	19.0	44.8	NS
Waist Circumference (cm)	105 (103; 107)	105	73	142	107 (104; 110)	106	78	137	102 (98; 105)	103	73	142	**
Waist-to-Height Ratio (-)	0.62 (0.61; 0.64)	0.62	0.44	0.93	0.61 (0.60; 0.63)	0.60	0.44	0.81	0.63 (0.61; 0.66)	0.64	0.46	0.93	NS
Mean of Five Skinfold Thicknesses (mm) ^2^	20.5 (19.6; 21.5)	20.8	7.2	35.2	18.8 (17.5; 20.0)	17.8	7.2	32.7	22.9 (21.5; 24.2)	23.4	8.1	35.2	***
	**n**	**%**			**n**	**%**			**n**	**%**			
Place of Residence Village Town < 500,000 City ≥ 500,000	13 21 109	9 15 76			7 12 63	9 15 77			6 9 46	10 15 75			NS.
Education: Primary Lower Secondary Higher Secondary Higher	14 45 59 25	10 31 41 17			3 33 32 14	4 40 39 17			2 15 33 11	3 25 54 18			NS.
Employment Status Unemployed Employed Retired Pensioner	5 48 65 25	3 34 19 18			3 33 32 14	4 40 39 17			2 15 33 11	3 25 54 18			***

^1^ Data were analysed after their logarithmic transformation. ^2^ Mean of five skinfold thicknesses: subscapular, biceps, triceps, abdominal, and supra iliac. ^3^ Level of significance for comparison of means between groups: ** *p* < 0.01; *** *p* < 0.001; NS—statistically insignificant differences. ^4^ Mean (95% confidence interval). ^5^ Median. ^6^ Minimum. ^7^ Maximum.

**Table 2:** The shares (%) of selected foods in the total antioxidant capacity (T-ORAC) of the daily diets in the study group. On average, four seasons ^1^.

Variable	T (n = 143) Men + Women				T(n = 82) Men				T (n = 61) Women				*p* ^2^
	X ^3^ (95%CI)	Me ^4^	Min. ^5^	Max. ^6^	X (95%CI)	Me	Min.	Max.	X (95%CI)	Me	Min.	Max.	
Total Drinks	32.9 (30.2; 35.7)	33.3	0.0	74.1	32.6 (28.8; 36.3)	32.1	0.0	74.1	33.5 (29.5; 37.4)	35.3	0.0	65.6	NS
Fruits	27.6 (25.3; 29.8)	25.6	1.9	82.0	27.8 (24.5; 31.0)	25.5	1.9	82.0	27.2 (24.0; 30.5)	26.0	4.8	56.2	NS
Vegetables, Incl. Potatoes	16.5 (15.2; 17.8)	15.0	3.9	46.5	15.7 (13.9; 17.4)	14.6	3.9	42.1	17.6 (15.6; 19.6)	15.7	6.3	46.5	*
Black Tea	14.4 (13.0; 15.8)	14.5	0.0	37.2	13.9 (11.9; 15.8)	14.0	0.0	37.2	15.1 (13.0; 17.2)	15.7	0.0	34.2	NS
Cocoa	13.3 (11.6; 14.9)	13.2	0.0	40.1	14.0 (11.8; 16.3)	13.5	0.0	40.1	12.2 (9.7; 14.7)	11.0	0.0	31.4	NS
Green Tea	5.3 (4.0; 6.5)	0.0	0.0	32.2	4.7 (3.1; 6.3)	0.0	0.0	25.8	6.1 (4.1; 8.2)	2.7	0.0	32.2	NS
Potatoes	5.2 (4.4; 6.0)	4.4	0.0	32.7	5.7 (4.5; 6.8)	5.0	0.0	32.7	4.6 (3.4; 5.8)	3.8	0.0	31.3	NS
Dry Fruits	3.4 (2.7; 4.1)	1.6	0.0	18.6	3.2 (2.3; 4.2)	1.1	0.0	15.9	3.7 (2.6; 4.8)	2.5	0.0	18.6	NS
Fruit Juices	2.7 (1.8; 3.6)	0.0	0.0	25.9	3.2 (1.9; 4.5)	0.0	0.0	25.9	2.0 (0.9; 3.1)	0.0	0.0	22.7	NS
Herbs and Spices	1.9 (1.5; 2.4)	0.9	0.0	16.8	1.4 (0.9; 2.0)	0.3	0.0	13.9	2.6 (1.9; 3.4)	2.0	0.0	16.8	*
Grains	1.1 (0.8; 1.5)	0.0	0.0	15.0	1.0 (0.5; 1.5)	0.0	0.0	15.0	1.3 (0.8; 1.9)	0.4	0.0	9.2	NS
Legumes	1.0 (0.6; 1.4)	0.0	0.0	13.3	1.1 (0.5; 1.7)	0.0	0.0	13.3	0.8 (0.3; 1.3)	0.0	0.0	10.3	NS
Nuts	0.9 (0.6; 1.2)	0.0	0.0	14.4	0.9 (0.4; 1.4)	0.0	0.0	14.4	0.8 (0.4; 1.2)	0.0	0.0	6.8	NS
Wines	0.5 (0.1; 1.0)	0.0	0.0	28.3	0.8 (0.0; 1.6)	0.0	0.0	28.3	0.2 (0.0; 0.3)	0.0	0.0	3.7	NS
Vegetable Juices	0.4 (0.2; 0.5)	0.0	0.0	6.0	0.4 (0.2; 0.6)	0.0	0.0	6.0	0.4 (0.2; 0.6)	0.0	0.0	4.5	NS
Red Wines	0.4 (0.1; 0.8)	0.0	0.0	22.2	0.6 (0.0; 1.3)	0.0	0.0	22.2	0.1 (0.0; 0.2)	0.0	0.0	2.6	NS
Oil from Olives	0.2 (0.1; 0.2)	0.0	0.0	1.9	0.1 (0.1; 0.2)	0.0	0.0	1.9	0.2 (0.1; 0.3)	0.0	0.0	1.4	NS
Chocolate in Total	0.04 (0.04; 0.11)	0.00	0.00	5.18	0.0 (0.0; 0.0)	0.0	0.0	0.0	0.1 (0.1; 0.3)	0.0	0.0	5.2	NS
Dark Chocolate	0.01 (0.01; 0.04)	0.00	0.00	1.99	0.0 (0.0; 0.0)	0.0	0.0	0.0	0.0 (0.0; 0.1)	0.0	0.0	2.0	NS

^1^ Data were analysed after their logarithmic transformation. ^2^ Level of significance for comparison of means between groups: * *p* < 0.05; NS—statistically insignificant differences. ^3^ Mean (95% confidence interval).^4^ Median. ^5^ Minimum. ^6^ Maximum.

**Table 3:** The shares (%) of selected foods in the total antioxidant capacity (T-ORAC) of the daily diets in the study group according to seasonality (mean; 95%CI) ^1^.

Variable	Spring ^2^	Summer	Autumn	Winter	*p* ^3^
T-ORAC (μmolTE/day)	19,915 (18,202; 21,628)	30,815 (28,260; 33,370)	24,120 (21,901; 26,338)	21,484 (19,564; 23,403)	***
Total Drinks	39.7 (36.3; 42.9)	27.1 (24.5; 29.7)	33.8 (30.7; 36.8)	36.2 (32.9;39.5)	***
Fruits	23.0 (20.3; 25.6)	42.1 (39.5; 44.7)	33.1 (30.3; 36.0)	27.0 (24.3; 29.6)	***
Vegetables, Incl. Potatoes	22.0 (20.2; 23.8)	18.9 (17.6; 20.3)	19.2 (17.3; 12.1)	20.1 (18.1; 22.2)	**
Black Tea	5.7 (3.9; 7.4)	19.8 (17.1; 22.4)	24.5 (21.3; 27.6)	26.3 (22.9; 29.6)	***
Cocoa	1.83 (0.51; 3.15)	1.4 (0.4; 2.3)	1.6 (0.4; 2.8)	1.7 (0.5; 3.0)	NS
Green Tea	2.8 (2.3; 3.3)	3.7 (2.5; 4.9)	0.01 (0.01; 0.03)	5.1 (3.5; 6.8)	NS
Dry Fruits	0.9 (0.4; 1.3)	0.5 (0.3; 0.6)	1.2 (0.8; 1.5)	0.8 (0.4; 1.3)	NS
Herbs and Spices	2.4 (1.9; 2.9)	1.5 (1.1; 1.8)	2.0 (1.5; 2.4)	2.2 (1.7; 2.7)	NS
Grains	0.5 (0.2; 0.8)	0.4 (0.2; 0.6)	0.5 (0.2; 0.7)	0.5 (0.2; 0.7)	NS
Legumes	0.4 (0.2; 0.6)	1.4 (1.2; 1.7)	0.4 (0.2; 0.6)	0.4 (0.2; 0.6)	NS
Nuts	1.8 (1.1; 2.5)	1.1 (0.7; 1.5)	1.5 (0.9; 2.0)	1.7 (1.0; 2.4)	NS
Wines	0.6 (0.1; 1.1)	0.4 (0.04; 0.8)	0.51 (0.1; 0.9)	0.5 (0.05; 1.0)	NS
Vegetable Juices	0.4 (0.2;0.5)	0.2 (0.1; 0.4)	0.3 (0.2; 0.5)	0.4 (0.2; 0.5)	NS
Red Wines	0.6 (0.05; 1.1)	0.4 (0.04; 0.8)	0.5 (0.1; 0.9)	0.5 (0.05; 1.0)	NS
Oil from Olives	0.2 (0.2; 0.3)	0.2 (0.1; 0.2)	0.2 (0.2; 0.3)	0.2 (0.1; 0.3)	NS
Chocolate in Total	8.7 (6.4; 11.1)	6.8 (5.0; 8.7)	8.1 (6.0; 10.3)	9 (6.4; 11.1)	NS
Dark Chocolate	3.2 (2.0; 4.4)	2.3 (1.4; 3.2)	2.5 (1.6; 3.4)	2.9 (1.8; 3.9)	NS

^1^ Data were analysed after their logarithmic transformation. ^2^ Level of significance for comparison of means between groups: ** *p* < 0.01; *** *p* < 0.001; NS—statistically insignificant differences. ^3^ Mean (95% confidence interval).

## Data Availability

The data presented in this study are available on request from the corresponding authors. These data are not publicly available due to privacy reasons.

## Supplementary Materials

The following are available online at: https://www.mdpi.com/article/10.3390/antiox12020292/s1, Table S1: The total dietary antioxidant capacity and nutritional value of daily diet in the study group. On average, in four seasons, values adjusted per 1000 kcal.

## Abbreviations

CVD	Cardiovascular disease
TAOX	Total dietary antioxidant capacity
WHO	World Health Organization
ORAC	Oxygen radical capacity
T-ORAC	Total diet antioxidant capacity (in μmolTE/day)
Q–ORAC	Total antioxidative density of diet (in μmolTE/1000 kcal)
