# Supplementary material for: The Total Dietary Antioxidant Capacity, Its Seasonal Variability, and Dietary Sources in Cardiovascular Patients
Source: Antioxidants (Basel). 2023 Jan 28;12(2):292. doi: 10.3390/antiox12020292 (PMC9952112; doi:10.3390/antiox12020292)
Supplement: Supplementary file 1 [file antioxidants-12-00292-s001.zip › antioxidants-2167397-supplementary.pdf]

Table S1. The total dietary antioxidant capacity and nutritional value of daily diet in the study group. On average. in four seasons. Values adjusted per 1000 kcal[1]

Variables	Men + women (n=143)				Men (n=82)				Women (n=61)				P[7]
	X[8] (95%CI)	Me[9]	Min.[10]	Max.[11]	X (95%CI)	Me	Min.	Max.	X (95%CI)	Me	Min.	Max.	
T-ORAC[7] (μmolTE/day)[2]	25 628 (23 254; 28 003)	23 983	5 064	90 931	25 174 (22 298; 28 050)	24 650	5 064	89 986	26 238 (22 138; 30 339)	23 758	6 956	90 931	NS
Q-ORAC$_{1000}$[3] (μmolTE/1000kcal)	14 386 (13 305; 15 467)	13 075	3 566	52 097	12 797 (11 784; 13 811)	12 323	3 971	25 702	16 521 (14 465; 18 576)	15 202	3 566	52 097	**
Energy (kJ)	4187	4187	-	-	4187	4187	-	-	4187	4187	-	-	-
Energia (kcal)	1000	1000	-	-	1000	1000	-	-	1000	1000	-	-	-
Total proteins (g/1000kcal)	40.3 (38.9; 41.6)	40.0	21.2	65.3	39.0 (37.2; 40.9)	38.2	21.2	63.3	41.9 (40.0; 43.8)	40.6	26.2	65.3	*
Plant proteins (g/1000kcal)	14.3 (13.8; 14.8)	14.4	7.6	26.5	13.7 (13.2; 14.3)	14.0	7.7	18.9	15.1 (14.2; 16.0)	15.1	7.6	26.5	**
Total fat (g/1000kcal)	44.2 (42.6; 45.7)	43.1	20.4	74.1	45.5 (43.6; 47.5)	45.2	27.1	68.2	42.4 (39.8; 44.9)	40.5	20.4	74.1	*
Total Carbohydrates (g/1000kcal)	123.3 (119.7; 126.8)	125.2	59.9	177.9	120.0 (115.7; 124.2)	122.2	72.6	160.5	127.7 (121.6; 133.8)	132.5	59.9	177.9	*
Sodium (mg/1000kcal)	1 045 (1 009; 1 080)	1 050	502	1 690	1 080 (1 032; 1 128)	1 064	614	1 690	998 (946; 1 050)	1 010	502	1 447	*
Potassium (mg/1000kcal)	1 871 (1 785; 1 958)	1 798	932	4 337	1 764 (1 680; 1 847)	1 719	983	2 998	2 016 (1 851; 2 180)	1 942	932	4 337	**
Calcium (mg/1000kcal)	817 (779; 854)	801	391	1 734	761 (723; 799)	771	391	1 246	892 (823; 960)	885	415	1 734	***
Phosphorus (mg/1000kcal)	695 (671; 720)	699	394	1 038	664 (631; 696)	631	397	932	738 (702; 774)	727	394	1 038	**
Magnesium (mg/1000kcal)	167 (159; 174)	164	92	300	159 (150; 168)	155	92	299	177 (165; 189)	178	92	300	**
Iron (mg/1000kcal)	6.1 (5.9; 6.4)	6.0	3.6	11.9	5.9 (5.6; 6.1)	5.6	3.8	10.2	6.5 (6.1; 6.8)	6.2	3.6	11.9	**
Zinc (mg/1000kcal)	6 (5; 6)	6	3	8	5.6 (5.3; 5.8)	5.5	3.6	8.3	6 (6; 6)	6	3	8	NS
Manganese (mg/1000kcal)	3.1	2.8	1.1	7.1	2.9	2.5	1.1	7.1	3.4	3.0	1.3	6.2	*

	(2.9; 3.4)				(2.7; 3.2)				(3.1; 3.7)				
Vitamin A (µg/1000kcal)	571 (516; 626)	487	142	3 084	477 (431; 522)	415	142	1 365	698 (590; 806)	603	301	3 084	***
Beta-carotene (µg/1000kcal)	2 534 (2 136; 2 931)	1 759	340	21 683	1 854 (1 540; 2 169)	1 389	340	8 280	3 446 (2 659; 4 234)	2 695	712	21 683	***
Vitamin D (µg/1000kcal)	1.3 (1.1; 1.4)	1.1	0.2	7.3	1.3 (1.1; 1.5)	1.1	0.3	5.3	1.3 (1.0; 1.6)	1.0	0.2	7.3	NS
Vitamin E (mg/1000kcal)	5.6 (5.3; 5.9)	5.4	2.4	14.0	5.3 (5.0; 5.6)	5.3	2.5	11.0	6.0 (5.5; 6.6)	5.6	2.4	14.0	**
Thiamine (mg/1000kcal)	0.6 (0.6; 0.7)	0.6	0.4	1.3	0.6 (0.6; 0.6)	0.6	0.4	0.9	0.7 (0.6; 0.7)	0.6	0.4	1.3	*
Riboflavin (mg/1000kcal)	0.9 (0.8; 0.9)	0.8	0.4	1.4	0.8 (0.8; 0.8)	0.8	0.5	1.2	0.9 (0.9; 1.0)	0.9	0.4	1.4	***
Niacin (mg/1000kcal)	9.2 (8.7; 9.7)	8.8	4.3	21.3	8.8 (8.2; 9.4)	8.6	4.3	17.0	9.6 (8.8; 10.5)	9.6	5.0	21.3	NS
Vitamin B6 (mg/1000kcal)	1.1 (1.0; 1.1)	1.0	0.6	2.4	1.0 (0.9; 1.0)	1.0	0.6	1.5	1.2 (1.1; 1.3)	1.2	0.6	2.4	***
Folate (µg/1000kcal)	130 (122; 138)	124	66	449	115 (108; 121)	108	66	202	151 (137; 166)	141	81	449	***
Cobalamin (µg/1000kcal)	1.8 (1.7; 2.0)	1.6	0.2	10.4	1.8 (1.6; 2.0)	1.6	0.5	6.5	2.0 (1.6; 2.3)	1.6	0.2	10.4	NS
VItamin C (mg/1000kcal)	47 (43; 52)	41	14	224	40 (37; 44)	37	15	99	56 (47; 65)	46	14	224	***
SFA[4] (g/1000kcal)	15.7 (14.9; 16.5)	15.2	5.5	29.2	16.3 (15.2; 17.4)	15.9	8.1	29.2	14.9 (13.7; 16.1)	14.9	5.5	26.6	NS
MUFA[5] (g/1000kcal)	18.2 (17.4; 19.0)	18.0	5.9	32.8	18.9 (17.9; 19.8)	19.1	8.8	28.6	17.2 (15.9; 18.6)	17.1	5.9	32.8	*
PUFA[6] (g/1000kcal)	7.5 (7.0; 7.9)	7.2	2.7	18.5	7.4 (6.9; 8.0)	7.4	2.7	16.8	7.5 (6.7; 8.2)	7.0	3.0	18.5	NS
Cholesterol (mg/1000kcal)	148 (139; 157)	139	30	345	150 (137; 164)	137	30	345	144 (132; 156)	139	38	321	NS
Sucrose (g/1000kcal)	13 (12; 14)	13	3	37	13 (12; 14)	12	3	31	14 (13; 16)	13	4	37	NS
Fibre (g/1000kcal)	14	12	5	36	12	11	6	23	16	14	5	36	***

	(13; 14)				(11; 13)				(14; 17)				

[1] Data were analysed after their logarithmic transformation. [2] Total antioxidant capacity of the daily diet. [3] Antioxidative density of the daily diet. [4] Saturated fatty acids. [5] Monounsaturated fatty acids. [6] Polyunsaturated fatty acids. [7] Level of significance for comparing means between groups: *p<0.05. **p<0.01. ***p<0.001. NS – statistically insignificant differences. [8] Mean (95% confidence interval). [9] Median. [10] Minimum. [11] Maximum.
